# Computed Tomography Evaluation of Normal Lacrimal Gland Dimensions in the Adult Pakistani Population

**DOI:** 10.7759/cureus.7393

**Published:** 2020-03-24

**Authors:** Shah Nawaz, Sajan Lal, Riffat Butt, Muhammad Ali, Bhesham Shahani, Arthina Dadlani

**Affiliations:** 1 Radiology, Dr. Ziauddin Hospital Clifton Branch, Karachi, PAK; 2 Radiology, Dr. Ziauddin Hospital, Karachi, PAK; 3 Radiology, Dr. Ziauddin University and Hospital, Karachi, PAK

**Keywords:** lacrimal gland, computed tomography

## Abstract

Objectives

To estimate the normal dimensions of lacrimal glands (LGs) in the Pakistani population using computed tomography (CT) scan and to determine the associations of LG dimensions with age, sex, and laterality.

Methods

The study population included subjects aged ≥16 years without orbital pathology who underwent CT evaluation of both the right and left LGs at Dr. Ziauddin University Hospital from June 2018 to November 2019. The mean axial length (AL), axial width (AW), coronal length (CL), and coronal width (CW) of each LG were measured separately.

Results

The 108 subjects enrolled in this study included 75 (69.4%) men and 33 (30.6%) women, with a mean age of 49±20 years. Right and left LG dimensions were similar, including mean AL (13.53±1.80 mm vs. 13.35±1.72 mm), mean AW (4.20±0.81 mm vs. 4.05±0.93 mm), mean CL (15.46±1.97 mm vs. 15.26±1.87 mm), and mean CW (3.99±0.80 mm vs. 3.86±0.81 mm). The AL of both LGs and the CL and CW of right but not left LGs were significantly lower in women than in men. Age showed significant correlations with the AL and CL of both LGs.

Conclusion

These findings establish the normal range of LG dimensions in adult Pakistani populations. Some LG dimensions are smaller in women than in men, as well as correlating with age.

## Introduction

The lacrimal glands (LGs) are paired, almond-shaped glands located in the lacrimal fossa area. The outer and upper areas of each orbit are located close to the superior and lateral rectus muscles [1]. LG diseases such as granulomatous tumors and autoimmune diseases can be confirmed by the presence of anomalies on orbital imaging [2]. LG function usually decreases with age, with the incidence of dry eye in subjects aged >65 years ranging from 15% to 25% [3-5]. Other conditions that can affect LGs, including their sizes, include granulomatous/autoimmune diseases and neoplastic disorders [2,6].

Although it is difficult to identify tumors by clinical examination alone, LG enlargement may be detected by physical examination. For example, pathological enlargement of the orbit may be present in 10% of these patients, including 27.7% of patients with ophthalmic sarcoidosis [7]. Advanced radiological techniques can accurately diagnose LG diseases, differentiating them from other orbital disorders, enabling optimal treatment, and determining patient responses to treatment [4,8].

Computed tomography (CT) scanning is regarded as the optimal radiological technique to accurately diagnose LG diseases [9]. CT scanning, especially axial and coronal CT scans, can accurately determine the borders of LGs, thus detecting enlarged or anomalous LGs. To determine LG abnormalities, however, it is necessary to determine the normal dimensions of LGs. The present study utilized CT scanning to measure LG dimensions in a normal Pakistani population and to determine the relationship between LG dimensions and age, sex, and laterality.

## Materials and methods

This study included subjects aged ≥16 years without orbital pathology who underwent CT evaluation of LGs at Dr. Ziauddin University Hospital from June 2018 to November 2019. The study protocol was approved by the hospital institutional review board, and each subject provided written informed consent.

All subjects underwent CT scanning of both the right and left LGs using a 16-slice CT scanner (Toshiba, Japan). Both coronal and axial views were taken of each LG. Axial and coronal lengths and widths of each LG were measured as described [10]. All images were evaluated by a consultant radiologist.

Axial length (AL) was measured from the extreme anterior to the extreme posterior tip and axial width (AW) was measured from the extreme medial to the extreme lateral edge. Coronal length (CL) was measured from the extreme superior to the extreme inferior tip and coronal width (CW) was measured from the extreme medial to the extreme lateral edge.

Data were analyzed using IBM SPSS Statistics for Windows, Version 24.0 (Armonk, NY: IBM Corp.). The normality of the data was evaluated using the Shapiro-Wilk test, with LG dimensions compared by independent sample t-tests. The association between LG dimensions and age was evaluated using Pearson correlation tests.

## Results

This study included 108 subjects, 75 (69.4%) men and 33 (30.6%) women, with a mean age of 49±20 years. The dimensions of right and left LGs, including mean AL, AW, CL, and CW were similar (Table 1). In contrast, there were significant differences in LG dimensions in men and women (Table 2). The ALs of both right (13.91±1.46 mm vs., 12.67±2.18 mm, p=0.001) and left (13.81±1.31 mm vs., 12.31±2.06 mm, p=0.001) were significantly longer in men than in women. Comparisons of right LGs showed that the mean CL (15.86±2.03 mm vs. 14.55±1.49 mm, p=0.001) was significantly greater and the mean CW (3.84±0.70 mm vs. 4.34±0.89 mm, p=0.002) was significantly lower in men than in women. In contrast, mean CL and CW of left LGs were similar in men and women, as were mean AW on both sides.

**Table 1:** Dimensions of the right and left lacrimal glands in men and women.

	Men (n=75)	Women (n=33)	P-value
Right lacrimal gland			
Axial length (AL), mm	13.91±1.46	12.67±2.18	0.001
Axial width (AW), mm	4.18±0.73	4.25±0.98	0.69
Coronal length (CL), mm	15.86±2.03	14.55±1.49	0.001
Coronal width (CW), mm	3.84±0.70	4.34±0.89	0.002
Left lacrimal gland			
Axial length (AL), mm	13.81±1.31	12.31±2.06	0.001
Axial width (AW), mm	3.99±0.87	4.17±1.05	0.35
Coronal length (CL), mm	15.38±1.99	14.97±1.55	0.29
Coronal width (CW), mm	3.85±0.73	3.86±0.97	0.95

**Table 2:** Dimensions of the right and left LGs. LG, lacrimal gland

	Right LG	Left LG	P-value
Axial length (AL), mm	13.53±1.80	13.35±1.72	0.45
Axial width (AW), mm	4.20±0.81	4.05±0.93	0.18
Coronal length (CL), mm	15.46±1.97	15.26±1.87	0.43
Coronal width (CW), mm	3.99±0.80	3.86±0.81	0.21

Several LG dimensions were found to correlate significantly with subject age (Table 3). The AL (r=-0.344; p<0.001) and AW (r=-0.491, p<0.001) of right LGs and the AL (r=-0.361, p<0.001) and AW (r=-0.498, p<0.001) of left LGs correlated significantly with age. In contrast, CL and CW of both right and left LGs did not correlate significantly with subject age.

**Table 3:** Correlation of age with dimensions of the lacrimal glands.

	Correlation co-efficient (r)	P-value
Right lacrimal gland		
Axial length (AL)	-0.344	<0.001
Axial width (AW)	-0.491	<0.001
Coronal length (CL)	-0.036	0.64
Coronal width (CW)	0.043	0.69
Left lacrimal gland		
Axial length (AL)	-0.361	<0.001
Axial width (AW)	-0.498	<0.001
Coronal length (CL)	-0.04	0.65
Coronal width (CW)	0.048	0.61

## Discussion

LGs are small glands located near the orbit but are frequently neglected during the evaluation of orbital diseases. Magnetic resonance imaging and CT scan are the two most used imaging methods for the evaluation of LG anatomy [11,12]. Determination of LG dimensions is very important because LG sizes are increased in many malignant and benign pathologic conditions [10]. The determination of LG size can help determine the risk of LG involvement. The present study evaluated LG dimensions in subjects in patients who underwent CT for evaluation of orbit pathology and were found to have normal LG.

The overall mean AL of LG glands in the present study was 13.44±1.76 mm, 13.53±1.80 mm for right LGs, and 13.35±1.72 mm for left LGs. The mean AL in this population was smaller than that of a Nigerian population, in which the mean ALs of right and left LGs were 14.6 and 14.5 mm, respectively [13]. In contrast, the mean CWs in this Pakistani population, 3.99 mm for right LGs and 3.86 mm for left LGs, were larger than those in the Nigerian population, 2.9 mm for right LGs, and 3.0 mm for left LG. The study in Nigerian subjects found no significant differences in LG dimensions between men and women patients. For right LGs, mean ALs were 14.46±2.04 and 14.76±1.87 mm, respectively; mean AWs were 4.17±0.46 and 4.08±1.41 mm, respectively; mean CLs were 20.96±3.08 and 20.31±3.23 mm, respectively; and mean CWs were 2.99±0.55 and 2.83±0.48 mm, respectively. Similar measurements were observed for left LGs [13]. 

A study in a Turkish population reported that the mean AL, AW, CL, and CW were 16.2, 4.1, 18.3, and 4.1 mm, respectively, for right LGs, and 16.0, 4.0, 18.3, and 4.1 mm, respectively, for left LGs [14]. In a Korean population, the mean AL, AW, CL, and CW were 14.7, 5.1, 17.7, and 5.2 mm, respectively, for right LGs, and 14.5, 4.8, 16.9, and 5.2 mm, respectively, for left LGs [15]. Detailed comparison of LGs dimensions with other populations is given in Table 4.

**Table 4:** Comparison of right and left lacrimal gland dimensions in different ethnicities.

	Present study	Turkish [14]	Nigerians [13]	Koreans [15]	Caucasians [10]
Right lacrimal gland					
Axial length (AL), mm	13.53	16.2	14.6	14.9	14.7
Axial width (AW), mm	4.20	4.1	4.1	4.1	5.1
Coronal length (CL), mm	15.46	18.3	20.7	20.9	16.9
Coronal width (CW), mm	3.99	4.1	2.9	3.6	5.2
Left lacrimal gland					
Axial length (AL), mm	13.35	16.0	14.5	14.7	14.5
Axial width (AW), mm	4.05	4.0	4.1	4.3	4.8
Coronal length (CL), mm	15.26	18.3	20.8	20.7	16.9
Coronal width (CW), mm	3.86	4.1	3.0	3.8	5.2

We found that age correlated significantly with ALs and AWs of both right and left LGs, but did not correlate significantly with CL and CW of both LGs. Similar outcomes were reported in a Nigerian population, with age showing a significant negative correlation with AL, AW, and CL, but a weak correlation with CW [13].

A major limitation of this study is that it was conducted in a single center with a small sample size, so our results may not reflect dimensions of LGs in the wider Pakistani population. Therefore, a large-scale multicenter study is needed to determine establish dimensions of LGs that are representative of the national population.

## Conclusions

Our finding that LG dimensions were smaller in a Pakistani population than in other populations suggests a need for large multicenter studies to determine normal LG dimensions in Pakistani subjects. Determination of the normal LG dimension may help in the diagnosis of conditions, such as orbital disorders, that alter LG size.
